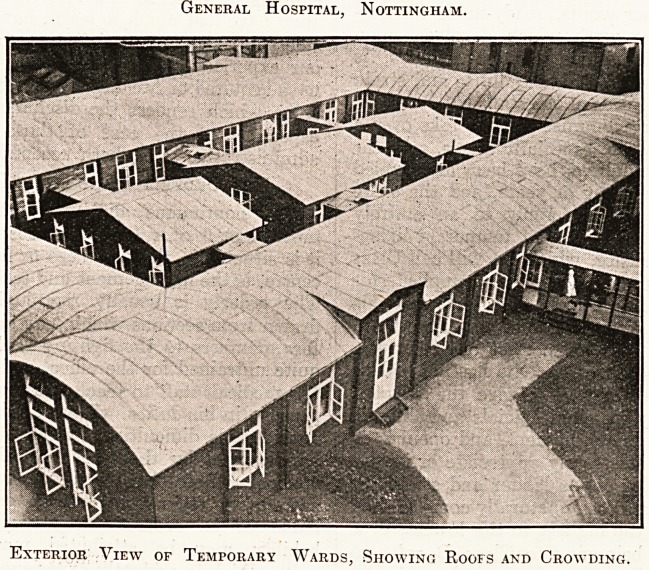# Hospital Provision: Its Difficulties and Limitations

**Published:** 1915-10-23

**Authors:** 


5th NORTHERN GENERAL HOSPITAL.

The Main Front and Gardens.

### THE WAR AND THE WOUNDED.

### HOSPITAL PROVISION.

### Its Difficulties and Limitations.

When the war broke out there was an immediate need to make provision for the reception of tens of thousands of wounded in suitable hospitals, where the severest, as well as the simpler, cases could be placed, and provided with the most modern and up-to-date facilities to secure the speedy recovery of the patients. This need was promptly supplied, a list of the available hospital accommodation having been given in The Hospital of August 15, 1914, page 553, and August 22, page 574.

The organisation throughout the United Kingdom for the reception of the sick and wounded from the war originated with Sir Alfred Keogh when Medical Director-General of the Army Medical Service. In 1907 he evolved a practical and extended plan which has steadily been developed. Under his direction steps were taken (*a*) for the establishment in selected buildings throughout the country of twenty-three general hospitals containing a minimum of 500 beds, (*b*) to set up and to make provision for an adequate medical and nursing staff locally for each such hospital. Many of the buildings selected had ample space surrounding them, so that in some cases it might be possible to provide in the adjacent grounds further hospital accommodation for from 1,000 to 1,500 extra patients. The selection of suitable buildings, mainly public buildings, was decided upon in substitution for the tent hospitals originally contemplated. The organisation of these hospitals has been carried out by the Royal Army Medical Corps, and also their equipment.

#### War Beds Originally Provided.

The hospital provision for the reception of the wounded from the war, so far as the Army is concerned, is entirely in the hands of the War Office. They originally relied upon three groups of hospitals which collectively could, if required, provide a total of 50,000 beds. There were first the military hospitals, which, if urgently necessary, could receive 20,000 wounded; next the Territorial hospitals just referred to, which, under similar conditions, could provide for 20,000 patients; and, lastly, the voluntary hospitals, which were estimated to maintain for the wounded 10,000 beds. It was originally estimated that these 50,000 beds would be ample, and possibly more than ample, to meet all the requirements of the wounded who were likely to arrive in the United Kingdom. At first it seemed that this estimate might prove correct, but as the war has proceeded, an urgent and ever-increasing demand has come for still further and greater hospital accommodation. The first result of this has been that the number of hospital beds made available in the three groups of hospitals just mentioned has been increased from 50,000 to about 60,000. No words that we can use would exaggerate the importance of the service which has been rendered by all the workers in every hospital where the wounded have been received, or by the voluntary hospitals, to the managers of which the Government owe a debt which we hope may one day be recognised and fully discharged.

#### First Estimates soon Exhausted.

As the war progressed and the number of wounded from the various centres in Flanders and France, Egypt and the Dardanelles, and elsewhere, increased, it quickly became apparent that the accommodation in the military hospitals, established and organised under the excellent scheme of Sir Alfred Keogh, combined with that placed at the disposal of the Government by the voluntary hospitals and the Poor-Law authorities, which collectively provided some 60,000 beds, must prove inadequate. There were in addition a large number of relief centres in private houses and elsewhere on offer which collectively amounted to several hundreds. Many of the latter have been brought into use as relief centres for convalescents during the war. An effort was then made to find suitable buildings in the larger centres of population throughout the country. This resulted in inquiries and the inspection of many buildings in populous centres by representatives of the Government, some of whom, though medical men, had no experience and only slight knowledge of the initial requirements which every building, to be effective, must fulfil, if it is to prove suitable for the reception and treatment of wounded men. From this cause much time was lost and trouble arose through the notification of a number of municipal and educational buildings which were not initially suitable, and which could never be made efficient for the purpose, however much money might be spent upon them.

### Business Methods Wanted.

We call attention to these circumstances because they illustrate how much in advance of the representatives selected by the central authorities in London to inspect buildings and place them on a list, which could be utilised by the War Office, the local and municipal authorities proved themselves to be as guides for securing additional beds for Navy and Army patients. Much criticism has been justly expended upon the lack of business methods pursued by the Government authorities in many matters, of which that for the selection of buildings for hospital purposes at Leicester constitutes an enlightening example. It once again demonstrates and enforces the wisdom of the demand many leading laymen have made for the introduction of business methods into Government offices. All kinds of buildings, apparently because they were large, were selected, including elementary schools and a huge concert-hall which Dr. Allison, who represented the Government, is reported to have approved for the purpose. As a matter of fact, the large concert-hall could not, under any conditions whatever, however much money might have been voted upon it, be adapted to provide suitable accommodation for hospital patients. In the case of school buildings, although it is possible that certain of them here and there might be adapted for the purpose of bed accommodation in the ward sense, there is almost necessarily a twofold objection to each. In the first place, the necessary adjuncts requisite to make an effective ward unit could only be provided by a huge expenditure, entailing not only an extravagant initial outlay, but a considerable cost when any school buildings selected should revert to the original purposes for which they were planned.

### Unsuitability of some School Buildings.

In the second place, the education of the children had to go on; it could not be entirely suspended for an indefinite period, and no one knew how long the war would continue. Besides, the taking of elementary school buildings for the purpose is a course which, in the best of circumstances, every capable educational and municipal authority must resist in the interests of the community which they represent. A further objection has been demonstrated at Nottingham, where some school buildings have been taken, and where the wards are reasonably suitable, but where also the accommodation for the kitchens and their attendant subsections are still incomplete, despite the money already expended, as to make it impossible for a hospital to be conducted in these buildings without the expenditure of still further sums, and a thorough rearrangement of, and addition to, the plans at present made. The fact is, in the case of school buildings there is seldom or never an adequate site available to provide for the accommodation of the nurses and other members of the staff, for the adequate kitchen provision, relaxation, and many other adjuncts which are essentials if the speedy recovery of the wounded and their welfare are to be the first considerations.

### How the Mistake at Leicester was Rectified.

The Leicester example illustrates the serious delays and difficulties which can be caused by central authorities commissioning someone without adequate technical knowledge to visit the local centres and select buildings. Fortunately for the wounded and for the Government, as well as for the taxpayers, Leicester citizens possess both backbone and commonsense, which led them to recognise on consideration that the majority of the buildings listed by the Government representative were utterly unsuitable for the purpose. They thereupon put their heads together and took infinite pains to find other and more suitable accommodation, with the result that to-day Leicester has, on the whole, some of the most excellent accommodation for the wounded to be found in any centre, at a cost which has been reduced to a minimum, whilst the standard of efficiency has been everywhere maintained at the highest point. This instance exhibits the value of men of business, who are also zealous citizens and patriots, when they are brought into the business of finding for the wounded necessary accommodation of the most suitable and adequate kind. In the result at Leicester not a single elementary school or its work—the discipline and teaching of its children—has been interfered with; the citizens still enjoy and benefit by the excellent administration of the large concert-hall—which is conducted by popular, enterprising men who enjoy the confidence of the people of Leicester. If Dr. Allison had been confined to his duties in the Metropolis, and the Leicester authorities had been asked at the outset to co-operate with the Government and make their own report as to the accommodation available, and to supply plans, time and money would have been saved, many difficulties would have been avoided, and everybody, from the Government downwards, would have been benefited.

[Continued on page 72.

### HOSPITAL PROVISION: ITS DIFFICULTIES AND LIMITATIONS (*continued*).

### The Part of the Local Authorities.

We hope, as still further beds and still more accommodation are needed for the wounded up and down the country, that those responsible in London will in future either employ only competent representatives, in the sense that they have adequate technical knowledge and have had experience of the requirements, possibilities, and limitations of the work to be done, or, better still, that they will throw the responsibility on the local authorities in conjunction with the leaders of medical opinion in each centre, reporting on the actual sites and accommodation which they regard as most suitable for the provision of hospital facilities and capable of being most readily and speedily put in a position to accommodate a good number of wounded men.

Here it may be well to indicate very briefly the initial requirements of every building and site which can properly be recommended for adaptation and use as a temporary hospital. It must contain a number of large halls or rooms, each adequately ventilated by cross-windows, or by artificial means or both, with a number of smaller rooms immediately adjacent to the larger ones. Each pavilion or block of buildings must be so planned and situated on the site as to afford the amplest circulation of air and the presence of abundance of light to every part of the building. There must be vacant spaces and unoccupied portions of the site which will permit an extension of the hospital provision when necessary, whereon additional offices and other necessary accommodation can be placed in temporary buildings of a suitable character, and these vacant portions of the site must be adjacent to the main buildings and capable of being readily connected with them without great cost or difficulty. Further, it is essential that the surrounding grounds or ground shall be amply sufficient to provide means of giving the wounded convalescent patients ample exercise and abundance of simple amusements, including sports and games. There must be accommodation, or it must be possible to provide accommodation in the building for a recreation hall, and provision must be made for the orderlies, in addition to a block for housing the nursing staff. Another essential requirement which experience has demonstrated more and more is that the site and grounds of every temporary hospital of the type we are dealing with shall be well isolated and self-contained, so that the wounded, as they approach nearer and nearer to complete recovery, shall be protected against the temptation of breaking bounds. To mention this requirement alone is to indicate at once the utter unsuitability of the overwhelming majority of elementary school buildings, or buildings used at present as places of amusement, and so to restrict the choice to a few often excellent buildings, which may be met with usually in most municipal centres and many of our counties, of the character of the disused buildings of the old asylum at Leicester, which we described in The Hospital of October 10, 1914.

To the public at large it is desirable to make known the importance of arousing local interest, so that every locality may move the authorities to schedule all suitable accommodation for the reception of the wounded within each district, and to follow the example of Leicester by approaching the central authorities.

3rd Northern General Hospital.

Exterior View, Showing Flat Roofs.

General Hospital, Nottingham.

Exterior View of Temporary Wards, Showing Roofs and Crowding.

## Figures and Tables

**Figure f1:**
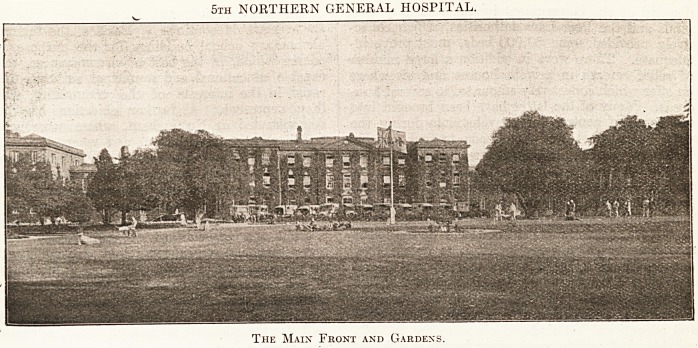


**Figure f2:**
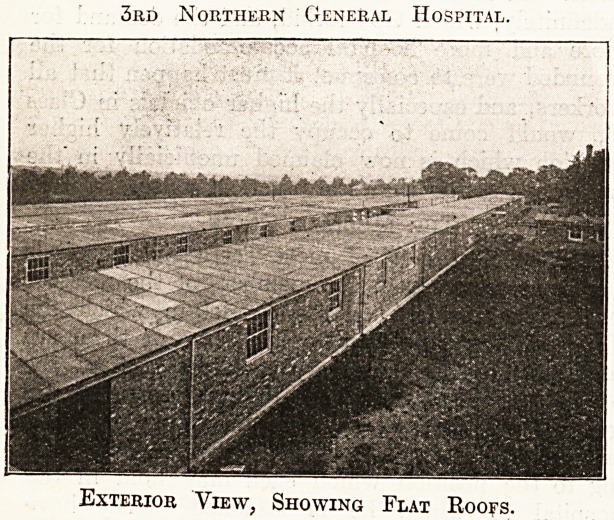


**Figure f3:**